# Effects of *Bothrops asper* Snake Venom on Lymphatic Vessels: Insights into a Hidden Aspect of Envenomation

**DOI:** 10.1371/journal.pntd.0000318

**Published:** 2008-10-15

**Authors:** Javier Mora, Rodrigo Mora, Bruno Lomonte, José María Gutiérrez

**Affiliations:** 1 Departamento de Parasitología, Universidad de Costa Rica, San José, Costa Rica; 2 Departamento de Microbiología, Universidad de Costa Rica, San José, Costa Rica; 3 Instituto Clodomiro Picado, Facultad de Microbiología, Universidad de Costa Rica, San José, Costa Rica; Liverpool School of Tropical Medicine, United Kingdom

## Abstract

**Background:**

Envenomations by the snake *Bothrops asper* represent a serious medical problem in Central America and parts of South America. These envenomations concur with drastic local tissue pathology, including a prominent edema. Since lymph flow plays a role in the maintenance of tissue fluid balance, the effect of *B. asper* venom on collecting lymphatic vessels was studied.

**Methodology/Principal Findings:**

*B. asper* venom was applied to mouse mesentery, and the effects were studied using an intravital microscopy methodology coupled with an image analysis program. *B. asper* venom induced a dose-dependent contraction of collecting lymphatic vessels, resulting in a reduction of their lumen and in a halting of lymph flow. The effect was reproduced by a myotoxic phospholipase A_2_ (PLA_2_) homologue isolated from this venom, but not by a hemorrhagic metalloproteinase or a coagulant thrombin-like serine proteinase. In agreement with this, treatment of the venom with fucoidan, a myotoxin inhibitor, abrogated the effect, whereas no inhibition was observed after incubation with the peptidomimetic metalloproteinase inhibitor Batimastat. Moreover, fucoidan significantly reduced venom-induced footpad edema. The myotoxic PLA_2_ homologue, known to induce skeletal muscle necrosis, was able to induce cytotoxicity in smooth muscle cells in culture and to promote an increment in the permeability to propidium iodide in these cells.

**Conclusions/Significance:**

Our observations indicate that *B. asper* venom affects collecting lymphatic vessels through the action of myotoxic PLA_2_s on the smooth muscle of these vessels, inducing cell contraction and irreversible cell damage. This activity may play an important role in the pathogenesis of the pronounced local edema characteristic of viperid snakebite envenomation, as well as in the systemic biodistribution of the venom, thus representing a potential therapeutical target in these envenomations.

## Author Summary

Envenomations by snakes of the family Viperidae (pit vipers) induce severe pathological alterations at the site of venom injection, such as edema, necrosis, hemorrhage, and blistering, which may lead to permanent tissue damage and disability. Edema is a prominent and common manifestation in these envenomations. The effect of viperid snake venoms in lymphatic vessels has not been previously investigated. This study analyzed the effect of the venom of *Bothrops asper*, the most important venomous snake in Central America, on the collecting lymphatic vessels of the mouse mesentery. The venom induced a rapid reduction in the lumen of these lymphatics, associated with a halting in the flow of lymph. These effects were reproduced by a myotoxic phospholipase A_2_ homologue isolated from this venom, but not by a hemorrhagic metalloproteinase nor by a coagulant serine proteinase. *B. asper* venom, and the purified myotoxin, were cytotoxic for smooth muscle cells in culture, thus suggesting that the alterations observed in lymphatics are due to the effect on smooth muscle cells of the lymphatic vessel wall. These results demonstrate a direct effect of *B. asper* venom on lymphatics, which is likely to contribute to the prominent edema characteristic of these envenomations.

## Introduction

Envenomations due to snakebites constitute a relevant, albeit neglected, health problem in many regions of the world, especially in rural areas of Africa, Asia, Latin America and Papua-New Guinea, where they have a high toll in terms of mortality and morbidity [1]–[4]. Envenomations by species classified in the family Viperidae, i.e. true vipers and pit vipers, and by some species of the family Elapidae, mostly cobras, are characterized by prominent local pathological effects which develop at the anatomical region where venom is injected [5],[6]. The rapid development of these local effects, together with the partial inability of antivenoms to neutralize them, often result in the appearance of permanent physical and psychological sequelae in these patients [2],[4],[7].

In Latin America, the vast majority of snakebite envenomations are inflicted by species of the genus Bothrops [6],[8],[9]. *Bothrops asper*, a species widely distributed in southern Mexico, Central America and the northern areas of South America [10], is responsible for the majority of cases in these regions [6],[11],[12]. Owing to the size of this species, and to the large volume of venom that it delivers, these envenomations involve a prominent local pathology which includes edema, hemorrhage, blistering, dermonecrosis and myonecrosis [6],[7],[9]. The pathogenesis of these pathological alterations has been investigated in experimental models. These effects result mostly from the combined action of Zn^2+^-dependent metalloproteinases (SVMPs) and myotoxic phospholipases A_2_ (PLA_2_) [7], [13]–[15]. SVMPs are able to degrade extracellular matrix components, such as those present in the basement membrane of microvessels and at the dermal-epidermal junction [16],[17], whereas myotoxic PLA_2_s disrupt the integrity of the plasma membrane of skeletal muscle fibers [15]. In addition, a prominent inflammatory reaction of multifactorial origin develops, resulting in a pronounced edema and an inflammatory cell infiltrate [6],[7]. Such edema, in turn, contributes to hypovolemia and may promote increments in intracompartmental pressures in some muscle compartments, thus inducing further ischemia and tissue damage [12],[18].

In spite of the advances in our understanding of venom-induced damage to muscle tissue and blood vessels, there is a gap in our understanding of the alterations occurring in lymphatic vessels as a consequence of these envenomations. The lymphatic vasculature plays a key role in the maintenance of fluid balance in the tissues, by removing fluid from the interstitial compartment [19]–[21]. The failure of lymphatics to fulfill this role, either by hereditary or acquired alterations, results in lymphedema [19], [20], [22]–[24]. In addition, lymphatics play critical functions in the immune surveillance and in the absorption of fat [20],[21]. In the case of snakebite envenomations, lymphatics contribute to the systemic absorption of venom toxins from the tissues [25]. Therefore, the study of potential effects of snake venoms on the structure and function of lymphatic vessels is relevant to understand the local pathology of these envenomations, and may have implications in terms of the pathogenesis of edema and the absorption of venom components from the tissues. The present study was designed to assess the effect of the venom of *B. asper* on the structure and function of mouse mesenteric collecting lymphatic vessels, and to identify which components in the venom are responsible for these alterations. Our results provide new insights into the understanding of toxin-mediated lymphatic flow impairment and delineate a possible involvement of these vessels in the pathophysiology of snakebite envenomation.

## Methods

### Venom and toxins

Venom was obtained from adult specimens of *B. asper* collected in the Pacific region of Costa Rica and kept at Instituto Clodomiro Picado. In some experiments, venom from neonate *B. asper* specimens (less than 30 days old) was also used. After collection, venom was lyophilized and stored at −20°C. Venom solutions were prepared immediately before the experiments, using 0.12 M NaCl, 0.04 M phosphate, pH 7.2 (PBS) as solvent. Myotoxin II, a phospholipase A_2_ homologue, was purified from the venom by cation-exchange chromatography on CM-Sephadex C-25, as previously described [26],[27]. Hemorrhagic metalloproteinase BaP1 was isolated by cation-exchange chromatography on CM-Sephadex C-25 and affinity chromatography on Affi-gel Blue, as described [28],[29]. A thrombin-like serine proteinase was isolated by benzamidine Sepharose affinity chromatography and anion-exchange chromatography on DEAE-Sepharose [30]. Homogeneity of the isolated toxins was assessed by sodium dodecyl sulfate (SDS)-polyacrylamide gel electrophoresis under reducing conditions [31].

### Intravital microscopy

Groups of five CD-1 mice (18–20 g) were anesthetized with a mixture of xylazine and ketamine, , and placed on a circulating water-heated bed. An abdominal mid section was made and the mesentery adjacent to a segment of small intestine was exposed. Then, the intestinal segment was positioned on a heated (37°C) brass device that surrounded an optical window for observing the lymphatic vessels [32]. The collecting lymphatic vessels selected for the study were those having an active flow of lymph. After several minutes of observation to ensure adequate lymph flow, the venom or isolated toxins, dissolved in 50 µL of PBS, were applied directly over the exposed intestinal segment. The doses used were 20, 50 and 100 µg for crude venom, 40 and 80 µg for myotoxin II, 40 and 100 µg for BaP1, and 10 µg for the thrombin-like enzyme. In the case of venom from neonate specimens, a dose of 100 µg was used. Toxin doses were selected on the basis of previous studies and were able to induce myonecrosis, hemorrhage and defibrination, respectively [30],[33],[34]. Controls were performed by applying 50 µL of PBS alone. A video was recorded for 15 min, and then snap shots were taken at 30 second intervals. At the end of the observation period, animals were sacrificed by an overdose of anesthetic. In some experiments, after 15 min of incubation, a portion of the mesentery, where lymphatic vessels were located, was dissected out, cut in small pieces and added to Karnovsky's fixative (2.5% glutaraldehyde, 2% paraformaldehyde in 0.1 M phosphate buffer, pH 7.2). Postfixation was performed with 1% osmium tetroxide. Afterwards, samples were dehydrated in ethanol and embedded in Spurr resin [35]. One micrometer sections were cut and stained with toluidine blue for histological observation. All animal experiments were approved by the Institutional Committee for the Use and Care of Animals (CICUA) of the University of Costa Rica.

### Measurement of the area of the lymphatic vessels

The changes in the relative area of the lymphatics in time were determined by quantification of the number of pixels corresponding to the lumen of those vessels from snap shots taken every 30 seconds. Image analysis was performed using a routine implemented in MATLAB (Mathworks) following morphological criteria and intensity threshold filtering. In each assay the initial area before the application of venom or toxins was considered as 100%, and the subsequent measurements were expressed in percentage relative to this 100%. The effects on the lymph flow were observed during 15 min after the application of the venom or isolated toxins, and the time when lymph flow in the selected lymphatic vessel stopped was recorded.

### Experiments with inhibitors

The peptidomimetic metalloproteinase inhibitor Batimastat (4-(*N*-hydroxyamino)-2R-isobutyl-3S-(thienylthiomethyl)-succinyl(-l-phenylalanine-*N*-methylamide; molecular mass: 478) was provided by British Biotech Pharmaceuticals, Ltd (Oxford, United Kingdom). All Batimastat suspensions were prepared by sonication in PBS containing 0.01% Tween 80 (PBS-Tween). The concentrations of Batimastat used were selected on the basis of our previous studies [36],[37]. A solution of 4 mg/mL of *B. asper* venom was incubated with an equal volume of 200 µM batimastat for 60 min at 37°C. Controls included venom incubated without Batimastat, and Batimastat incubated without venom. Inhibition of venom metalloproteinases was demonstrated by assessing the elimination of venom hemorrhagic activity in mice [37]. The polyanionic polysaccharide fucoidan (molecular mass: 135 kDa; Sigma–Aldrich, St. Louis, MO, USA) was used to inhibit myotoxic PLA_2_s. Solutions of *B. asper* venom were incubated with fucoidan at an inhibitor: venom weight ratio of 3∶1 for 60 min at room temperature. Controls included venom incubated without fucoidan, and fucoidan incubated without venom. Inhibition of myotoxic activity of PLA_2_s in these conditions was corroborated in mice by determining the plasma creatine kinase (CK) activity in plasma [38],[39].

### Inhibition of edema-forming activity by fucoidan

Edema-forming activity induced by venom and myotoxin II was assessed by measuring the increment in the footpad thickness with a low pressure spring caliper [40]. The ability of fucoidan to inhibit venom and myotoxin-induced edema was evaluated by incubating either venom or myotoxin with fucoidan, for 60 min at room temperature, at an inhibitor: venom weight ratio of 3∶1 or at an inhibitor: toxin weight ratio of 10∶1 (which corresponds approximately to a molar ratio of 1∶1). Controls included venom or toxin incubated with PBS alone. Mice (18–20 g; n = 5) were then injected subcutaneously, in the right foot pad, with 50 µL of solutions containing either venom or toxin alone, or venom or toxin incubated with fucoidan. The doses injected were 5 µg, in the case of venom, and 6 µg, in the case of myotoxin. These doses were selected on the basis of previous studies since they induce a submaximal edema response [41],[42]. The left foot pad was injected with 50 µL of either PBS alone or fucoidan alone. At various time intervals (15 and 30 min, and one, three and six hr), the thickness of both footpads was determined. Edema was expressed as the percentage increment in the thickness of the right footpad as compared to the left one.

### Cytotoxicity assays on smooth muscle cells

The human umbilical artery smooth muscle cell line (HUASMC, Cell Applications, Inc., San Diego, CA) was used. Smooth muscle cells were grown in 25 cm^2^ bottles using Endothelial Cell Growth Medium (Cell Applications, San Diego, CA, USA), in a humidified atmosphere with 7% CO_2_, at 37°C. Subconfluent monolayers were treated, for 5 min at 37°C, with trypsin (1500 U/mL), containing 5.3 mM EDTA. Resuspended cells were seeded in 96-well microplates, at an approximate initial density of 1–4×10^3^ cells per well, in the same culture medium. Cytotoxicity was assessed by determining the release of the cytosolic enzyme lactic dehydrogenase (LDH), as previously described [43],[44]. Briefly, smooth muscle cell cultures were incubated with various concentrations of Myotoxin II, dissolved in culture medium, at a volume of 100 µL/well. Aliquots of the supernatant in culture wells were collected at 3 hours. LDH activity was determined by using a commercial kit (Biocon LDH-P, Analyticon Biotechnologies AG, Germany). Reference controls for 0% and 100% cytolysis consisted of medium alone and medium from cells incubated with 0.1% (v/v) of Triton X-100, respectively. All assays were carried out in triplicates.

For inhibition studies, smooth muscle cells were incubated with either crude *B. asper* venom or with *B. asper* venom previously incubated, during 60 min at room temperature, with fucoidan. A constant amount of fucoidan was incubated with various concentrations of venom, and a ratio of fucoidan∶venom of 3∶1 (w∶w) was achieved at the highest concentration of venom used. After incubation, 100 µL of solutions, containing various doses of venom, were added to cell culture wells, followed by 3 hr incubation. LDH activity in the medium was determined as described. All assays were performed in triplicates.

### Analysis of permeability to propidium iodide in smooth muscle cells

Smooth muscle cells were cultured as described. Culture wells were then washed with PBS, followed by the addition of cell culture medium containing 1% fetal calf serum (FCS), 1 µg/mL propidium iodide (PI) and Myotoxin II (20 µg/well). Hoechst-33258 (1 µg/mL; Sigma-Aldrich) was added to identify all cell nuclei in the preparation. Cells were incubated at various time intervals, and observed in a fluorescence microscope. This methodology allowed the detection of plasma membrane damage [45].

### Statistical analyses

The significance of the differences between pairs of means was determined by the Student's *t* test, with a p value of 0.05 determining the limit of significance. When more than two groups were compared, an analysis of variance was performed, followed by a Tukey test.

## Results

### Intravital microscopy observations: Crude venom reduces lymphatic vessel lumen and impairs lymphatic flow

**Figure 1:**
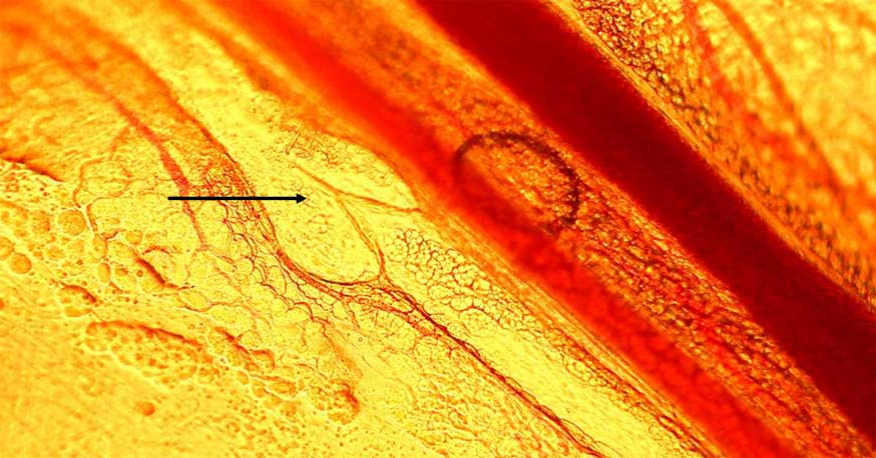
Light micrograph of a collecting lymphatic vessel in a mouse mesentery preparation, observed by intravital microscopy. A characteristic valve is present (arrow). 200×.

**Figure 2:**
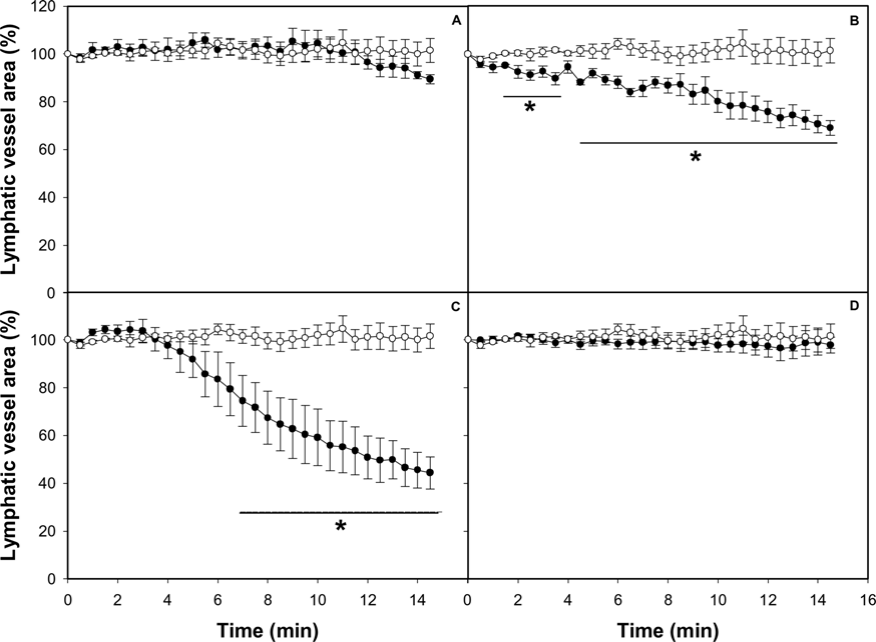
Effect of *B. asper* venom on the caliber of mouse mesentery collecting lymphatic vessels. The mesentery was exposed as described in Methods, and 50 µL of either PBS or various concentrations of *B. asper* venom, dissolved in 50 µL PBS, were applied onto the preparation. A video was recorded before venom application and then during 15 min after venom addition. Snap shots were taken at 30 second intervals. A section of the lymphatic was selected and the area of its lumen determined by an image analysis program for each snap shot. Changes in the area were expressed as percentage, considering 100% the area of the lymphatic vessel lumen before the application of venom or PBS. Results are presented as mean±S.E.M. of five different preparations. Open circles represent control samples treated with PBS alone and closed circles represent samples treated with venom (A: 20 µg, B: 50 µg, C: 100 µg of venom from adult specimens, D: 100 µg of venom from neonate specimens). *p<0.05 when compared with control preparations treated with PBS alone.

**Figure 3:**
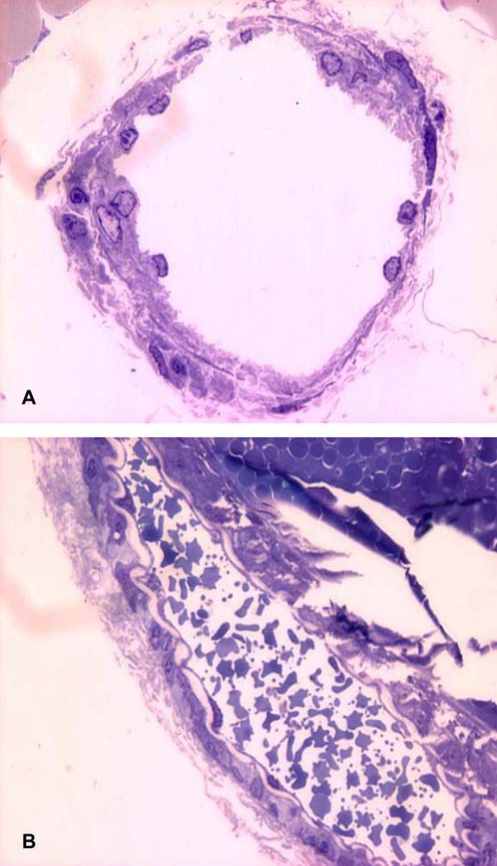
Histological alterations in lymphatic vessels of mouse mesentery preparations treated with *B. asper* venom. PBS (A) or 100 µg *B. asper* venom (B) were applied onto mouse mesentery, as described in Methods. After 15 min, preparations were dissected out and processed for histological observation. Notice the presence in B of abundant erythrocytes within the lymphatic vessel lumen, as a consequence of the hemorrhagic effect of the venom. Toluidine blue staining. 1,000×.

In order to study the effects of crude venom on lymphatic vasculature, collecting lymphatics, located in the mesentery and presenting the characteristic valves, were identified using intravital microscopy (Fig 1). For analysis of the effect of venom, only collecting lymphatics having a regular lymph flow were used. Application of PBS alone did not induce any effect in the lumen of these vessels, as evidenced by the maintenance of a constant area in the lumen (Fig 2). When venom was applied onto the mesentery, a dose-dependent effect was observed, characterized by a significant contraction of the vessel wall, resulting in a reduction of the lumen; such effect occurred within 2–5 min of application of venom doses of 50 and 100 µg, but not with the dose of 20 µg (Fig 2A, B and C). The effect remained at the end of the 15 min observation period. When using doses of 50 and 100 µg, a complete halting of lymph flow occurred between 5 and 7 min after application of venom (Table 1). When using 50 µg of venom, there was a restart of lymph flow at later time intervals, whereas with the dose of 100 µg the flow was not recovered (not shown). In addition, a prominent hemorrhage developed within the first 5 min of application of the venom, evidenced by abundant areas of extravasation. Many erythrocytes were observed flowing through the lymphatics at early time periods, before the halting of lymph flow. Histological observations corroborated the presence of erythrocytes inside lymphatics (Fig 3). When a dose of 100 µg venom of neonate *B. asper* specimens was tested, there was no effect on the lumen of lymphatic vessels nor halting in lymph flow (Fig 2D), but a prominent hemorrhage developed in the mesentery.

**Table 1:** Time required for the complete halting of lymph flow in collecting lymphatics after application of *B. asper* venom or purified toxins.

Treatment	Time required for halting of lymph flow (min)
PBS	-*
20 µg of *B. asper* venom	-
50 µg of *B. asper* venom	7.30±0.99
100 µg of *B. asper* venom	5.50±0.40**
100 µg *B. asper* venom treated with Batimastat	6.05±0.87
100 µg *B. asper* venom treated with fucoidan	-
100 µg of BaP1	-
10 µg of thrombin-like enzyme	-
40 µg of MT-II	6.70±0.82
80 µg of MT-II	5.30±0.60**

*: (-) indicates that lymph flow was not halted during the 15 min of observation.

**: p<0.05 when compared with results obtained after application of 50 µg of *B. asper* venom. Values represent mean±S.D. (n = 5).

### Myotoxin II reproduces the effect of crude venom on lymphatic vessels

**Figure 4:**
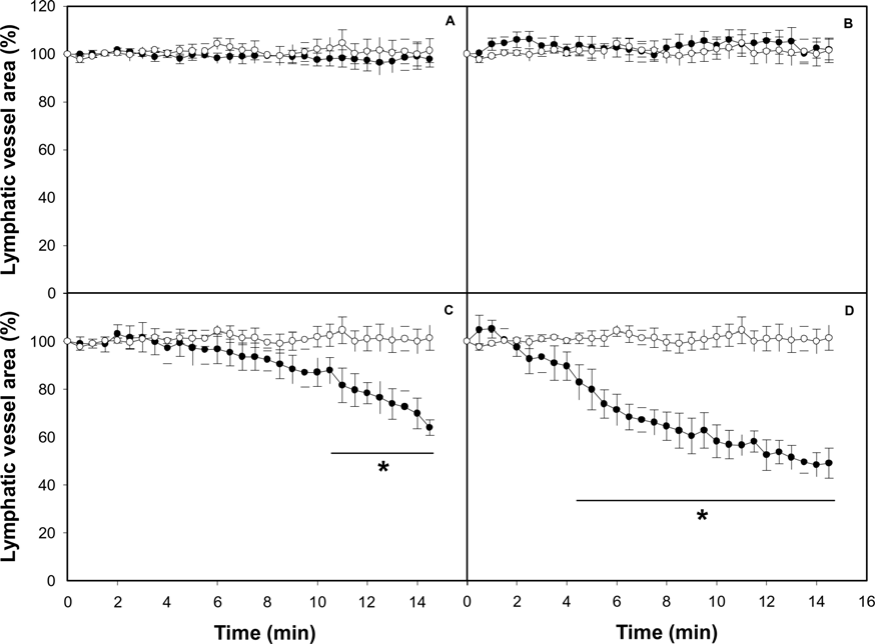
Effect of isolated toxins on the caliber of mouse mesentery collecting lymphatic vessels. The mesentery was exposed as described in Methods, and 50 µL of either PBS or different toxins isolated from *B. asper* venom, dissolved in 50 µL PBS, were applied onto the preparation. Observations were performed as described in the legend of Fig 2. Changes in the area were expressed as percentage, considering 100% the area of the lymphatic vessel lumen before the application of toxins or PBS. Results are presented as mean±S.E.M. of five different preparations. Open circles represent control samples treated with PBS alone and closed circles represent samples treated with toxins: (A) 100 µg of hemorrhagic metalloproteinase BaP1; (B) 10 µg of coagulant thrombin-like serine proteinase; (C) 40 µg of the phospholipase A_2_ homologue myotoxin II; (D) 80 µg of myotoxin II. *p<0.05 when compared with control preparations treated with PBS alone.

To determine which component of the venom is responsible for the activity on the lymphatic vessels, single isolated toxins were tested. Application of BaP1, a hemorrhagic metalloproteinase, and of a thrombin-like coagulant serine proteinase isolated from *B. asper* venom did not induce any effect in the lumen of lymphatics (Fig 4 A,B), at doses inducing widespread hemorrhage, in the case of BaP1, and defibrination, in the case of the thrombin-like enzyme. In contrast, the PLA_2_ homologue myotoxin II reproduced the effects induced by crude venom in lymphatics, since it induced a dose-dependent reduction in the lumen of these vessels (Fig 4 C, D) and a concomitant halting in the lymph flow (Table 1).

### A myotoxin inhibitor abolishes the effect of the venom on lymphatic vessels

**Figure 5:**
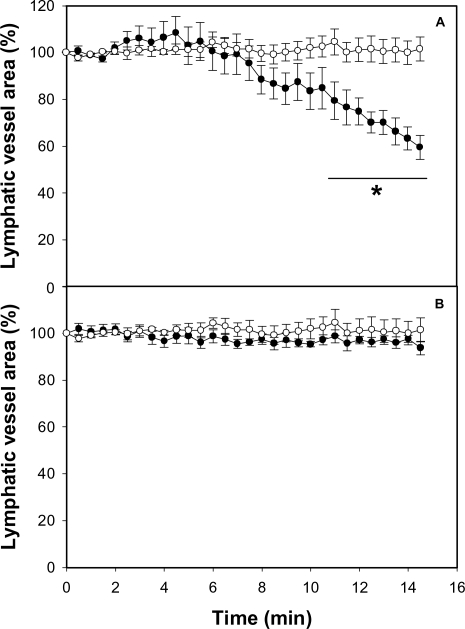
Effect of metalloproteinase inhibitor Batimastat and myotoxin inhibitor fucoidan on the activity of *B. asper* venom on mouse mesentery collecting lymphatics. Before application, venom solutions were incubated with either Batimastat or fucoidan, as described in Methods. The mesentery was exposed and 50 µL of either PBS or the mixtures of 100 µg venom and inhibitors, dissolved in 50 µL PBS, were applied onto the preparation. Observations were performed as described in the legend of Fig 2. Changes in the area were expressed as percentage, considering 100% the area of the lymphatic vessel lumen before the application of venom or PBS. Results are presented as mean±S.E.M. of five different preparations. Open circles represent control samples treated with PBS alone and closed circles represent samples treated with venom incubated with Batimastat (A) or fucoidan (B). *p<0.05 when compared with control preparations treated with PBS alone.

To confirm that involvement of specific venom components in the effect of crude venom on lymphatics, the ability of several inhibitors to block the effect of *B. asper* venom was assessed. Incubation of *B. asper* venom with the metalloproteinase inhibitor Batimastat resulted in complete abrogation of hemorrhagic activity, without preventing the lymphatic alterations induced by the venom (Fig 5A). In contrast, incubation of venom with fucoidan, a known inhibitor of myotoxic PLA_2_s, completely abolished the effect of venom on the lymphatics (Fig 5B), without affecting its hemorrhagic activity. Control preparations in which Batimastat or fucoidan alone were applied to mesentery did not show any observable effect (not shown).

### A myotoxin inhibitor significantly reduces edema-forming activity of venom

**Figure 6:**
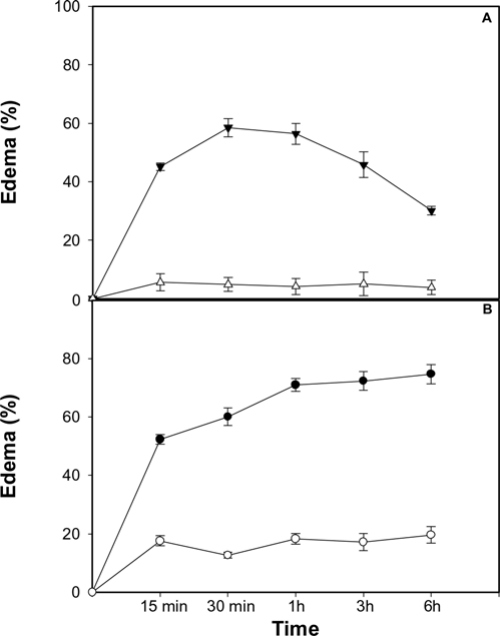
Inhibition of edema-forming activity of myotoxin II and *B. asper* venom by fucoidan. Myotoxin II (A) or venom (B) were incubated with fucoidan, and edema was assessed at various time intervals after subcutaneous injection in the footpads of mice, as described in Methods. Edema was expressed as the percentage of increment in the thickness of the footpad as compared with the contralateral footpad that was injected with either PBS or fucoidan alone. Closed triangles and circles correspond to edema induced by myotoxin or venom alone, whereas open triangles and circles correspond to edema induced by myotoxin or venom incubated with fucoidan. Results are presented as mean±S.E.M. (n = 5). Edema was significantly reduced (p<0.05) by fucoidan at all time intervals tested.

In order to assess whether inhibition of myotoxin has an effect on edema-forming activity, venom or myotoxin II were incubated with fucoidan and edema was tested in the mouse footpad model. Fucoidan completely abolished edema-forming activity of myotoxin II (Fig 6A). Moreover, fucoidan markedly reduced the edema-forming activity of *B. asper* venom (Fig 6B), thus evidencing that myotoxins play a significant role in the pathogenesis of edema induced by this venom.

### Myotoxin II induces cytotoxicity in smooth muscle cells

**Figure 7:**
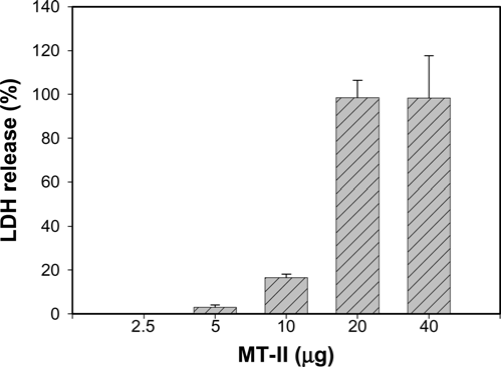
Cytotoxic activity of *B. asper* myotoxin II on smooth muscle cells in culture. Cells were seeded in 96-well microplates, at an approximate initial density of 1–4×10^3^ cells per well, and were incubated with various concentrations of myotoxin II, dissolved in culture medium, at a volume of 100 µL/well. Aliquots of the supernatant in culture wells were collected at 3 hr and LDH activity was determined, and expressed as percentage LDH release. Reference controls for 0% and 100% release consisted of medium alone and medium from cells incubated with 0.1% (v/v) of Triton X-100, respectively. Results are presented as mean±S.E.M. (n = 3).

**Figure 8:**
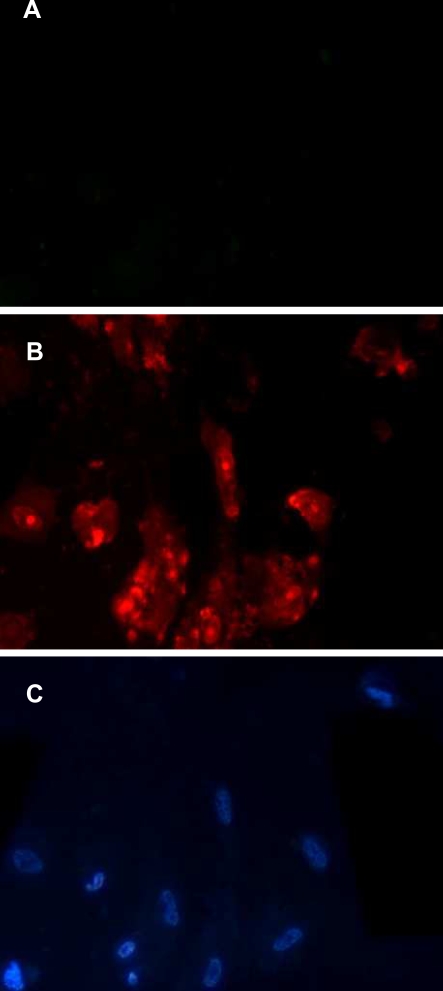
Plasma membrane disruption of smooth muscle cells by the action of myotoxin II. Cells, seeded as described in the legend of Fig 7, were incubated with myotoxin II (20 µg per well in cell culture microplates) and 1 µg/mL of propidium iodide (PI), both dissolved in cell culture medium containing 1% FCS. (A) Cells incubated with medium alone, without toxin, showing no penetration of PI. (B) Cells incubated with myotoxin, evidencing penetration of PI after 5 min of incubation. (C) The same microscopic field of (B), stained with Hoechst-33258 to visualize the nuclei of all cells. 400×.

**Figure 9:**
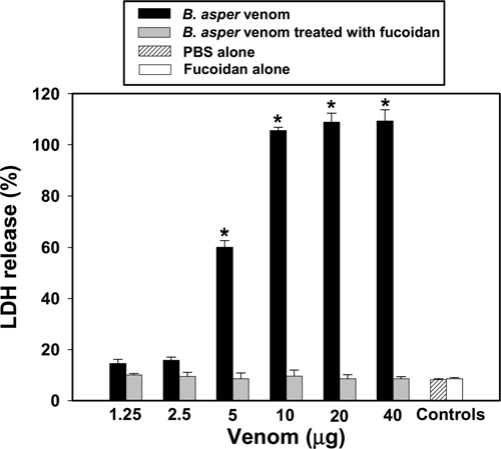
Inhibition of the cytotoxic activity of *B. asper* venom on smooth muscle cells in culture. Venom was incubated with fucoidan for 60 min at room temperature, and cytotoxicity was tested as described in Methods and in the legend of Fig 7. Controls included PBS alone, venom incubated with PBS, and fucoidan alone. The dose-dependent cytotoxic effect of venom, reflected in LDH release, was completely abrogated by incubation with fucoidan. Results are presented as mean±S.D. (n = 3). * p<0.05 when compared with cells incubated with venom+fucoidan.

In order to gain insights into the mechanism through which *B. asper* venom affects lymphatics, cultured smooth muscle cells were treated with myotoxin II. Myotoxin induced a rapid and dose-dependent cytotoxicity, evidenced by the release of the cytosolic enzyme LDH (Fig 7). The toxin also induced a rapid increment in the permeability of plasma membrane to PI (Fig 8) which corroborated the disruption in the integrity of plasma membrane. Moreover, incubation of *B. asper* venom with fucoidan, prior to its addition to smooth muscle cell culture completely abrogated cytotoxicity in these cells (Fig 9), thus confirming the relevance of basic myotoxins in this effect.

## Discussion

Lymphatic vessels play a key role in the fluid and pressure balances in tissues, by regulating fluid homeostasis. This function is performed by two main types of vessels, i.e. the initial lymphatics and the collecting, or contractile, lymphatics [46],[47]. The initial lymphatics are blind-ending vessels constituted by an endothelial cell lining, characterized by a discontinuous basement membrane, and are devoid of pericytes and smooth muscle cells [21],[46],[48]. Collecting lymphatics are characterized by the presence of a smooth muscle cell layer which exhibits periodic contractions regulated by transmural pressure, luminal lymph flow, neural input and humoral factors [46],[48],[49]. Such contractile activity depends on the regulation of Ca^2+^ fluxes in muscle cells [50]. A system of valves is present in collecting lymphatics, allowing the directional fluid transport along these vessels through functional units known as lymphangions [46],[47],[49]. Our results demonstrate a conspicuous effect of *B. asper* venom on the collecting lymphatics of the mouse mesentery.

An intravital microscopy methodology was used, coupled to an image analysis system, to follow the morphological alterations in lymphatics, to quantify the extent of reduction in their lumen, and to assess the halting of lymph flow. It allowed the determination of the area of a segment of a lymphatic vessel, which is directly proportional to the lumen of the vessel. The most notorious observation upon the local application of *B. asper* venom was a dose-dependent reduction in the lumen of the vessel, with a concomitant halting in the flow of lymph. Such effects had a rapid onset and were prolonged for the 15 min of observation. Interestingly, in most cases, the reduction in lymphatic vessel diameter preceded the halting of lymph flow. Experiments performed with toxins isolated from this venom showed that these effects were reproduced by a myotoxic PLA_2_ homologue, but not by a hemorrhagic metalloproteinase or by a coagulant serine proteinase. The lack of involvement of metalloproteinases in the pathogenesis of this effect was further corroborated by the inability of Batimastat, a potent metalloproteinase inhibitor [37], to abrogate the effect. Metalloproteinases are responsible for the main alterations induced by viperid venoms in the microvasculature, resulting in capillary disruption and hemorrhage [16],[51].

Furthermore, our observations showed that the venom of neonate specimens of *B. asper* does not induce lymphatic vessel damage. There are drastic differences in the composition of venoms from neonate and adult specimens of *B. asper* [52],[53]. Venom of neonates has a high concentration of metalloproteinases and, consequently, a higher hemorrhagic activity than venom of adults. In contrast, venom of neonates is devoid of myotoxic PLA_2_s [52],[54], whereas myotoxins are abundant in the venom of adults [52],[54],[55]. The lack of effect on lymphatics of strongly hemorrhagic and weakly myotoxic neonate *B. asper* venom further supports a key role of basic myotoxic PLA_2_s and PLA_2_ homologues, and the lack of involvement of hemorrhagic metalloproteinases, in the pathogenesis of lymphatic vessel damage.

The lack of effect of metalloproteinase on lymphatics may be related to the fluid pressure within these vessels. It has been observed that *B. asper* SVMPs do not directly affect the integrity of endothelial cells in culture [34],[56],[57]. Therefore, the drastic ultrastructural alterations induced in capillary endothelial cells in vivo are likely to depend on the biophysical hemodynamic forces operating in vivo, which promote distension and rupture of endothelium after the initial weakening of capillary wall structure provoked by the SVMP-induced cleavage of basement membrane components [51],[58]. Therefore, in a low-pressure system, such as that of lymphatic vessels, the degradation of basement membrane components would not result in the distension and disruption of vessel integrity. In addition, the presence of a muscle cell layer surrounding endothelium in the collecting lymphatics provides a mechanical protection to the effect of basement membrane degradation. The role of a thrombin-like coagulant enzyme was assessed in order to test whether clotting of fibrinogen in the lymph would affect lymph flow. Results clearly evidenced that coagulant and procoagulant components, which are common in viperid snake venoms [59] and have been described in *B. asper* venom [30],[60], are not responsible for the effect of venom on lymphatics.

The pathological effects induced by *B. asper* venom on lymphatics were reproduced by myotoxin II, a myotoxic PLA_2_ homologue. Furthermore, the key role played by this type of component was corroborated by the complete elimination of the effect when venom was preincubated with fucoidan, an inhibitor of myotoxic PLA_2_s [38],[39]. *B. asper* venom, and many other viperid venoms, contain a group of highly cationic PLA_2_s and PLA_2_ homologues which are responsible for skeletal muscle damage, i.e. myonecrosis [15]. Many of these myotoxins, such as the one used in this study, are catalytically-inactive variants, known as Lys49 PLA_2_s, able to disrupt membranes and to induce myonecrosis [61]. Our observations provide evidence, for the first time, that myotoxic PLA_2_s are also capable of damaging smooth muscle cells with a dose-dependency range similar to that previously described for skeletal muscle myotubes in culture [44]. It is suggested that myotoxin II affects smooth muscle cells by disrupting the integrity of the plasma membrane, thus promoting a Ca^2+^ influx and, at cytotoxic concentrations, the release of the cytosolic enzyme LDH and the influx of PI. Such membrane perturbation, with the concomitant increment in cytosolic Ca^2+^ concentration, would promote muscle cell contraction [49],[50] and the consequent reduction in lymphatic vessel lumen. The sustained contraction of lymphatic smooth muscle precludes the process of cyclic contractility, characteristic of normal lymphatic vessels, which promotes lymph pumping [49]; as a consequence, the flow of lymph is halted. Furthermore, a sustained increment in cytosolic Ca^2+^ would trigger a series of degenerative events that provoke irreversible cell damage, i.e. necrosis [15]. In this scenario, lymphatic smooth muscle cells would be irreversibly damaged and, therefore, the vessel would be functionally impaired since lymph flow requires a coordinated tonic and phasic contraction of smooth muscle in collecting lymphatics [49]. Hence, although our intravital observations allowed the observation of acute changes only, the observed cytotoxicity on smooth muscle cells suggests that venom-induced lymphatic damage is likely to be long lasting, with evident pathophysiological implications. Besides the cytotoxic, i.e. pathological effect of myotoxins in lymphatic vessel smooth muscle cells, the observed contraction may be also due to the PLA_2_-induced release of inflammatory mediators [62], some of which may promote smooth muscle contraction.

Our results shed light into a hitherto unknown aspect of snake venom-induced local pathology which may have a relevant pathophysiological role. Viperid snakebite envenomations are characterized, among other features, by a prominent edema of rapid onset which is very difficult to manage therapeutically [63]. Pharmacological studies have identified a number of inflammatory mediators involved in this edema-forming activity of *Bothrops* sp venoms [41],[62],[64]. The described effect of *B. asper* venom on collecting lymphatics highlights another possible mechanism for the pathogenesis of edema, since the alterations described are likely to affect the lymph flow in envenomated tissues. Our observation that the myotoxin inhibitor fucoidan drastically reduces venom-induced edema supports the hypothesis that basic myotoxic PLA_2_s and PLA_2_ homologues play a key role in the pathogenesis of edema by this venom. Thus, the edematogenic action of myotoxic PLA_2_s may be due to a combination of their effect on lymphatic vessels and their ability to release inflammatory mediators in the tissue [42],[62].

Our findings may also explain previous observations showing that, at low venom doses, there is a transient edema that peaks early on after injection and then declines [40], whereas when higher venom doses are administered, swelling has an extended time-course [40],[41]. It is suggested that such prolonged edema is the consequence of the pathological alterations described in lymphatic vessels, with the consequent impairment in lymph formation and interstitial fluid balance. Since local edema may complicate local pathology by promoting increments in interstitial pressure in muscle compartments, leading potentially to compartmental syndrome-associated ischemia and necrosis, lymphatic vessel damage is a significant component of snake venom-induced pathology that deserves consideration at experimental and clinical settings. Our results also suggest that inhibition of the cytotoxic action of venom myotoxins in smooth muscle cells may be of therapeutic benefit in snakebite envenomation.

## Supporting Information

Alternative Language Abstract S1 Translation of the Abstract into Spanish by José María Gutiérrez
(0.02 MB PDF)
