# Supplementary material for: Effects of Bothrops asper Snake Venom on Lymphatic Vessels: Insights into a Hidden Aspect of Envenomation
Source: PLoS Negl Trop Dis. 2008 Oct 15;2(10):e318. doi: 10.1371/journal.pntd.0000318 (PMC2563035; doi:10.1371/journal.pntd.0000318)
Supplement: Alternative Language Abstract S1 — Translation of the Abstract into Spanish by José María Gutiérrez (0.02 MB PDF) [file pntd.0000318.s001.pdf]

Spanish translation of Abstract provided by José María Gutiérrez

Efecto del veneno de la serpiente *Bothrops asper* en los vasos linfáticos: análisis de un aspecto desconocido del envenenamiento.

Antecedentes. Los envenenamientos causados por la serpiente *Bothrops asper* constituyen un problema de salud serio en Centroamérica y partes de Sudamérica. Estos envenenamientos se caracterizan por una patología local severa, que incluye el desarrollo de un edema prominente. Dado que el flujo linfático desempeña un papel central en el mantenimiento del balance de fluidos en los tejidos, en este trabajo se estudió el efecto del veneno de *B. asper* en los vasos linfáticos colectores.

Metodología y hallazgos principales*:* Se aplicó el veneno de *B. asper* a preparaciones de mesenterio de ratón y se observaron los efectos mediante el empleo de una metodología de microscopia intravital acoplada a un programa de análisis de imágenes. El veneno de *B. asper* indujo una contracción dosis-dependiente de los vasos linfáticos colectores, lo cual redundó en una reducción del lumen de dichos vasos y en una interrupción del flujo de la linfa. Este efecto se reprodujo cuando se aplicó un homólogo de fosfolipasa A_2 (PLA_2) miotóxico aislado de este veneno, pero no cuando se aplicó una metaloproteinasa hemorrágica y una serina proteinasa coagulante tipo-trombina. En concordancia con estos hallazgos, la incubación del veneno con fucoidan, un inhibidor de miotoxinas, eliminó por completo el efecto, en tanto no ocurrió inhibición cuando el veneno se incubó con Batimastat, un inhibidor peptidomimético de metaloproteinasas. Además, el fucoidan redujo significativamente el efecto edematígeno del veneno en el modelo de pata de ratón. El homólogo miotóxico de PLA_2, el cual induce necrosis de músculo esquelético, fue citotóxico sobre células de músculo liso en cultivo celular e indujo un incremento de la permeabilidad de estas células al ioduro de propidio.

Conclusiones y relevancia: Nuestros resultados muestran que el veneno de *B. asper* afecta los vasos linfáticos colectores del mesenterio mediante la acción de PLA_2s miotóxicas sobre las células de músculo liso de la pared vascular, induciendo contracción y daño celular irreversible. Este efecto puede jugar un papel importante en la patogénesis del edema que caracteriza los envenenamientos por vipéridos, así como en la biodistribución sistémica del veneno, representando por lo tanto un posible blanco terapéutico relevante en estos envenenamientos.

Palabras clave: Veneno de serpiente, *Bothrops asper*, fosfolipasas A_2 miotóxicas, metaloproteinasas, linfáticos, edema.
